# Correction: Randomized Phase II Trial of Dendritic Cell/Myeloma Fusion Vaccine with Lenalidomide Maintenance after Upfront Autologous Hematopoietic Cell Transplantation for Multiple Myeloma: BMT CTN 1401

**DOI:** 10.1158/1078-0432.CCR-24-2408

**Published:** 2024-10-01

**Authors:** David J. Chung, Nina Shah, Juan Wu, Brent Logan, Lina Bisharat, Natalie Callander, Giulia Cheloni, Kenneth Anderson, Thinle Chodon, Binod Dhakal, Steve Devine, Poorvi Somaiya Dutt, Yvonne Efebera, Nancy Geller, Haider Ghiasuddin, Peiman Hematti, Leona Holmberg, Alan Howard, Bryon Johnson, Dimitra Karagkouni, Hillard M. Lazarus, Ehsan Malek, Philip McCarthy, David McKenna, Adam Mendizabal, Ajay Nooka, Nikhil Munshi, Lynn O’Donnell, Krina Patel, Aaron P. Rapoport, Jane Reese, Jacalyn Rosenblatt, Robert Soiffer, Dina Stroopinsky, Lynne Uhl, Ioannis S. Vlachos, Edmund K. Waller, James W. Young, Marcelo C. Pasquini, David Avigan

In the original version of this article (1), author Krina Patel is mistakenly omitted from the author list. The error has been corrected in the latest online HTML and PDF versions of the article and the author contributions and disclosures have been updated as necessary. The authors regret the error.
